# Sustained Delivery of IL-1Ra from PF127-Gel Reduces Hyperglycemia in Diabetic GK-Rats

**DOI:** 10.1371/journal.pone.0055925

**Published:** 2013-02-08

**Authors:** Muhammad Sajid Hamid Akash, Kanwal Rehman, Hongying Sun, Shuqing Chen

**Affiliations:** 1 Institute of Pharmacology, Toxicology and Biochemical Pharmaceutics, College of Pharmaceutical Sciences, Zhejiang University Hangzhou, China; 2 College of Pharmacy, Government College University Faisalabad, Pakistan; University of Milan, Italy

## Abstract

Interleukin-1beta (IL-1β) is a major cause for induction of various inflammatory mechanisms that are decisively involved to provoke pathogenesis of type 2 diabetes mellitus (T2DM). Interleukin-1 receptor antagonist (IL-1Ra) a naturally occurring anti-inflammatory antagonist of IL-1β has been recently approved for treatment of T2DM but due to its short half-life, higher doses and frequent dosing intervals are required. Pluronic F-127 (PF127) has previously shown to prolong the release of various proteinous drugs and their serum half-lives. Subsequently, in our previous work, we developed a new dosage form of IL-1Ra using PF127 and investigated its in-vitro and in-vivo effects. Here in present work, we have extended this approach using diabetic Goto-kakizaki (GK) rats. We administered IL-1Ra loaded in PF127 gel subcutaneously for one month into GK rats. IL-1Ra loaded in PF127 gel exhibited a sustained and prolonged hypoglycemic effects on treated animals. Intraperitoneal glucose tolerance test (IPGTT) results showed that IL-1Ra loaded in PF127 gel increased glucose tolerance along with increased insulin sensitivity and β-cell’s secretory function in treated rats. Moreover, significant reduction in pro-insulin/insulin ratio, lipid profiles and interleukin 6 (IL-6) were also observed. Immunohistochemical analysis showed slight macrophages infiltration in pancreatic islets. Histochemical analysis revealed no PF127-induced alteration in the normal physiology of skin and kidney of treated animals. Hence, we concluded that IL-1Ra loaded in PF127 gel has potential to exhibit broad spectrum anti-inflammatory effects alleviating the symptoms of T2DM.

## Introduction

Diabetes mellitus is one of the leading causes of mortality and is considered among top 5 fatal diseases that may cause diabetic microvascular (retinopathy, nephropathy, and neuropathy) [1] and macrovascular (coronary artery, cerebrovascular, and peripheral vascular disease) complications [2]. Recently, inflammation has attained considerable attention in pathogenesis of diabetes mellitus due to which it has been recently considered as chronic auto-inflammatory syndrome [3], [4] in which various inflammatory mechanisms are involved [5]. One of the decisive reasons for the progression of diabetes mellitus is glucoliptoxicity that is induced by inadequate secretion of insulin from β-cells of pancreatic islets and/or by the impairment of glucose uptake in peripheral tissues [6], [7]. Abnormally elevated levels of glucose and free fatty acids (FFAs) may impart their cytotoxic effects on β-cells of pancreatic islets [8] through the activation of various pro-inflammatory mediators [9]. Once these pro-inflammatory mediators are activated, they may not only damage the β-cells but can also diminish the potential capacity of remaining β-cells to secrete adequate amount of insulin according to the requirement of blood glucose in both types of diabetes [10]. On the other hand, these potentiated pro-inflammatory mediators may also initiate inflammation in peripheral tissues impairing glucose uptake [9] and may lead to insulin resistance. Hence, insulin resistance is associated with the state of low grade-inflammation [11], [12] and has been considered as a hallmark of T2DM. Once insulin resistance is developed, the circulating levels of glucose and/or FFAs become alarmingly high and by entering the β-cells of pancreatic islets they may induce the production of IL-1β along with other IL-1-dependent cytokines and chemokines [9], [13], [14].

Among these pro-inflammatory mediators, IL-1β is a master pro-inflammatory cytokine that plays a decisive role in the progression of T2DM. It regulates the inflammatory processes in various tissues [15]. Augmented levels of glucose and some associated metabolic derangements (dyslipidemia and/or insulin resistance) may lead to hyperglycemia, which further potentiate the production of IL-1β from β-cells [16], [17]. Nevertheless, IL-1β is considered the major cause for defective insulin secretion, β-cell apoptosis and/or insulin resistance in peripheral tissues, this further suggests that inflammation may be directly involved in the pathogenesis of diabetes mellitus [5], [9], however, anti-inflammatory therapeutic modalities may help prevent inflammation [18], [19]. IL-1Ra is a naturally occurring anti-inflammatory antagonist of IL-1β that competitively binds with interleukin-1 receptor-I (IL-1RI) [20], [21] and protects β-cells from glucolipotoxicity-induced functional impairment and apoptosis. Various studies have been conducted in which therapeutic effects of IL-1Ra are observed using diet-induced diabetic mice [22], GK rats [19], cultured human’s pancreatic islets [16], [23] and human with T2DM [18], [24]. IL-1Ra has the ability not only to block the synthesis of IL-1β [25] but also of IL-1-dependent cytokines and chemokines [17], [19], [23]. Although, IL-1Ra has been approved for the treatment of T2DM [18], [22] but short biological half-life (6–8 hrs) of IL-1Ra has been a major hinder for its therapeutic efficacy causing complexity in the dosage adjustment and frequency of drug administration.

Taking into account, the broad-spectrum therapeutic potentials of IL-1Ra, recently in our previous work, we developed a sustained delivery system for IL-1Ra based on Food and Drug Administration (FDA) approved biodegradable polymer PF127 as thermosensitive gel [26]. In aqueous solutions, PF127 has thermoreversible gelation property and extends the stability of proteins [26]–[29] with their complete recovery when dissolved at body temperature [30]. Depending upon the concentration used, at room temperature, PF127 exists as a solution and can be easily administered via parenteral route. After administration, it rapidly converts into rigid-semisolid gel network at body temperature [26]. Previously, it has already been used for the sustained delivery of various proteins [31]–[35].

Here, in our present work, we have extended our previous approach by using diabetic male GK and/or wistar rats to study the sustained release effects of IL-1Ra from PF127 gel. We used 4 weeks old GK rats and wistar rats as a control. GK rats are specially developed from wistar rats after their repetitive inbreeding [36] selected at the upper limit of normal glucose tolerance. In GK rats, reduction in β-cell mass occurs during their fetal development followed by mild hyperglycemia that appears post weaning at the age of 3–4 weeks after birth [37] which impairs the ability of β-cells to secrete insulin in response of increase glucose. In our present study, we administered PF127 gel having IL-1Ra for one month and then investigated the effects of IL-1Ra on different metabolic parameters. Moreover, we have also focused on for any adverse events of PF127 relating to kidney function and at the site of drug administration.

## Materials and Methods

### Animals

Experimental studies were performed on fed, age-matched male GK and non-diabetic wistar rats. Diabetic GK rats were obtained from Academy of Medical Science, Zhejiang, China. Non-diabetic Wistar rats were used as controls. Animals were weaned on day 28, with water *ad libitum,* fed with commercial chows in a temperature, humidity and light (12 hrs cycles) controlled room. All animal experiments were conducted in accordance with the accepted standards for animal care approved by laboratory of animal centre, Zhejiang University Hangzhou, China.

### Preparation of 25% PF127 Gel and In-vivo Treatment of IL-1Ra

The PF127 (Zhejiang Hisun Pharmaceutical Co., China) gel was prepared as previously described [26]. Prior to the subcutaneous administration of IL-1Ra into the loose skin over the shoulder/neck of GK rats, IL-1Ra (10 mg/kg body weight of rat) was mixed with 200 µl of pre-sterilized 25% PF127 gel. Treatment was initiated 2–3 days following weaning (4 weeks old), after onset of mild fed hyperglycemia [38] and was continued up to 4 weeks. Thereafter, nonfasting glycemia was determined with glucose analyzer (AccuChek Active, Roche Diagnostics, USA) 2–3 times per week at 9–10 am. At the end of one month treatment, for a merciful killing, the rats were anesthetized with pentobarbital. Blood was collected from abdominal vein in a sterile syringe (5 ml capacity) with hypodermic needle (21 G). Serum was separated from the collected blood by centrifugation (5,000 RPM, 20 min) at 4°C. The serum was stored at −20°C until further analysis. Skin tissues and kidneys were collected to perform H&E staining. A separate tissue of pancreas was also collected to perform Immunohistochemistry. All the tissue samples were preserved in 10% formalin solution until further analysis.

### Intraperitoneal Glucose Tolerance Test (IPGTT)

IPGTT was carried out at the last week of treatment in nonanesthetized animals. Glucose (Sinopharm Chemical reagent Co., China) solution was injected into animals (2 mg/kg body weight) after an overnight starvation. Blood was then collected from the tail 5 minutes before injection, as well as after every 15, 30, 60, and 120 min of injection for the measurement of serum insulin whereas; at each time-point glucose was measured by a glucometer [39].

### Insulin Sensitivity Determinations

Homeostasis model assessment (HOMA) for insulin resistance index (HOMA-IR) was calculated as follows; 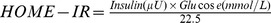 and insulin secretion [*β*-cell function (HOMA-*β*)] was calculated as; 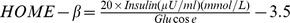 [40]. Fasting insulin/fasting glucose ratio (FIGR) was calculated as; 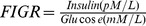 [41]. Quantitive insulin sensitivity check index (QUICKI) was calculated as; 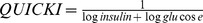 [42]. The serum levels of glucose and insulin were estimated under fasting conditions.

### Biochemical Analysis

Serum insulin and pro-insulin were analyzed using rat insulin and pro-insulin ELISA kit (Mercodia AB, Sweden). Serum FFAs were assayed using Autosera enzymetic colorimetric assay NEFA (Sekisui Medical Co., Japan). Serum levels of urea nitrogen (UN), uric acid (UA), triglycerides (TGs) and total cholesterol (TC) were measured by enzymatic assay (Beckman coulter, USA). Serum creatinine, high density lipoproteins (HDL), low density lipoproteins (LDL) and very low density lipoproteins (VLDL) were measured by enzymatic assay (Autec Diagnostica, Germany). Serum level of IL-6 was measured by using rat IL-6 ELISA Quantikine kit (R&D System, Inc., USA). All serum parameters were measured under fed conditions.

### Urine Analysis

Urine was collected from all rats in metal free propylene tubes for 24 hrs. After collection, urine samples were centrifuged at 5000 RPM for 15 minutes at 4°C to remove cells and other debris material before further analysis. 24 hrs urinary albumin and creatinine was measured by using rat nephelometry (Beckman Coulter, USA) and enzymatic assay (Autec Diagnostica, Germany) respectively. The urinary albumin/creatinine ratio (ACR) was calculated from these two measurements.

### Immunohistochemistry

Immunohistochemistry was performed for mouse monoclonal antibody CD68 (Abcam) with isolated pancreatic islets from sacrificed rats. Staining was visualized using peroxidase coupled secondary antibody with subsequent incubation. Antibody-stained surface area of pancreatic islets taken by single blinded observer using Olympus color video camera with Histolab software (Biocom) was visualized.

### Histochemical Analyses

Skin tissues and kidneys of sacrificed rats were separated and fixed in 10% formalin solution. After fixing, the processing of skin and kidney tissues was carried out with different percentages of ethanol and then embedded in paraffin wax for 6 hrs. Thin slices of processed skin and kidneys were separated by microtoming which were then fixed on glass slides with gelatin and kept in oven for 12 hrs at 58°C. Finally, these slides were treated with hematoxylin and eosin (H&E) and observed for histochemical alterations in these tissues. Images were taken by single blinded observer using Olympus color video camera with Histolab software (Biocom).

### Statistical Analysis

Data were expressed as mean ± SD with the number of individual experiments elucidated. Whole data were analyzed using non-linear regression analysis using GraphPad prism 5. The level of significant difference was assessed using student’s *t*-test and analysis of variance (ANOVA) with Newman-Keuls posthoc test for multiple comparison analysis. The level of significant difference was set at P<0.05 in the target parameters between groups.

## Results

### IL-1Ra Reduces the Extent of Hyperglycemia in GK Rats

**Figure 1:**
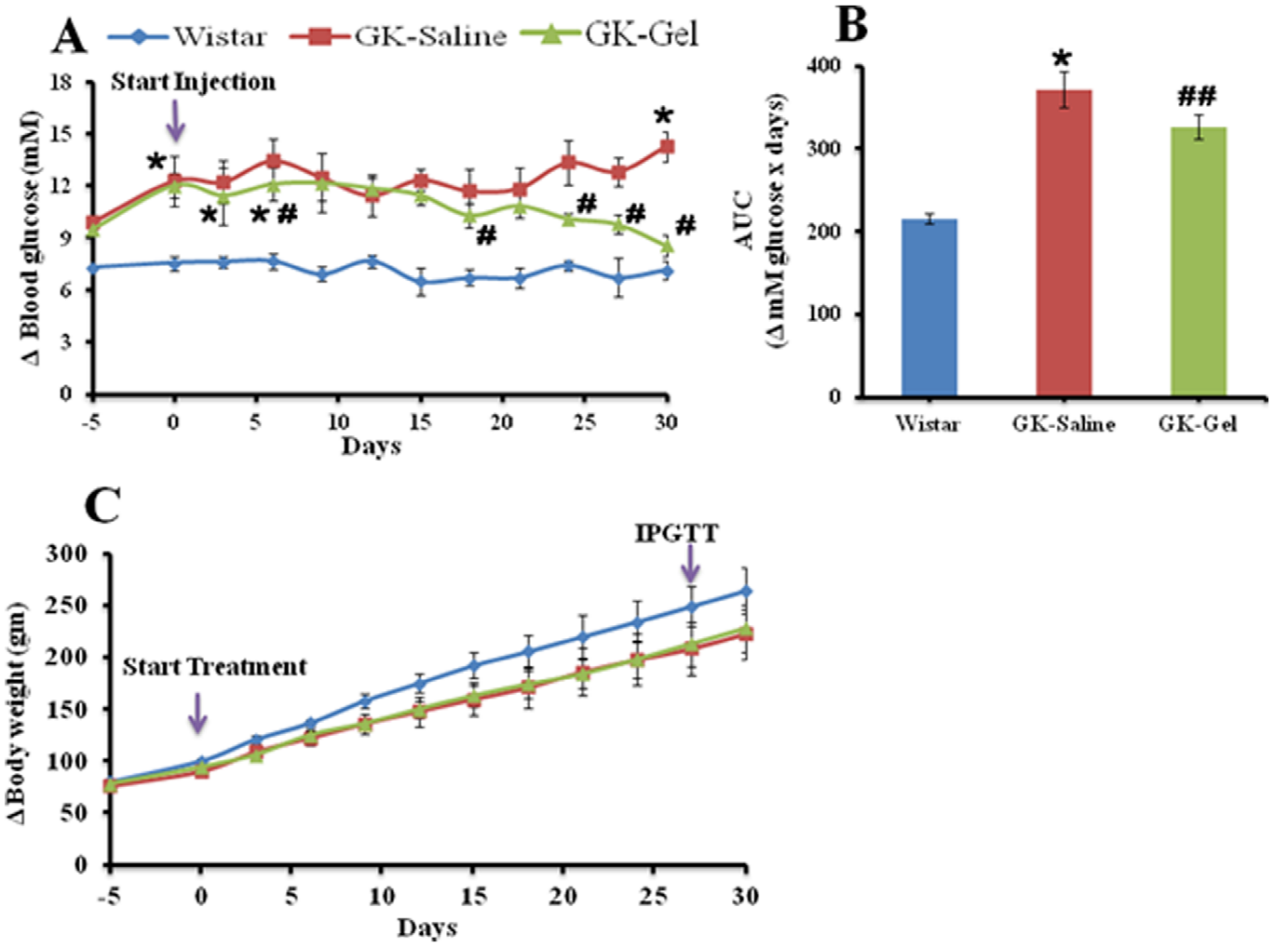
Sustained delivery of IL-1Ra from 25% PF127 gel prevents the extent of hyperglycemia in diabetic GK-rats. Treatment was started in 4 weeks old male wistar and GK-rats. 200 µl of NS was administered in wistar (n = 5) and GK (n = 5) rats once a day whereas, IL-1Ra (10 mg/kg body weight) in 200 µl presterilized PF127 gel in GK (n = 5) rats. Animal groups had different blood glucose values when the treatment was started (see text). IL-1Ra administration and measurement of blood glucose was performed at 9–10 am. (A) Delta (Δ) fed blood glucose, (B) AUC for Δ fed blood glucose values over 4 weeks of treatment, and (C) Δ bodyweight during treatment are shown. “n” represent the total number of animals in each group. ^*^, P<0.05; ^**^, P<0.01; compared to wistar rats groups. ^#^, P<0.05; ^##^, P<0.01 compared to GK-Saline group.

To assess therapeutic potentials of IL-1Ra on nonobese diabetic GK rats, we administered IL-1Ra (10 mg/kg body weight of rat) loaded in 25% PF127 gel once daily via subcutaneous injection for 4 weeks. In this study, we used age-matched one month old GK and wistar rats. GK rats have the ability to develop mild hyperglycemia post weaning at the age of 3–4 weeks [37]. On the day of start of treatment, the values of fed plasma glucose were 7.56±0.42 mM for wistar rats, 12.30±1.24* mM for GK-saline and 12.00±0.68* mM for GK-Gel (*, P<0.05 vs. wistar rats) whereas, their body weights were 100.20±1.85 gm, 90.30±1.86 gm and 94.00±1.94 gm respectively. IL-1Ra loaded in PF127 gel showed reduction in fed hyperglycemia (Fig. 1A and B) with no effect on body weight during treatment period (Fig. 1C).

Throughout the treatment period, we measured fed plasma glucose levels 2–3 times/week, initially, no significant difference (P<0.05) was observed between GK-Saline vs. GK-Gel but a clear significant difference (P<0.05) was noticed among GK-Saline and GK-Gel vs. wistar rats. After 15 days of continuous sustained delivery of IL-1Ra through PF127 gel, a significant difference (P<0.05) in the values of fed plasma glucose appeared between GK-Saline vs. GK-Gel till the last day of treatment. At the end of treatment, glucose values under fed conditions for wistar rats, GK-Saline and GK-Gel were 7.08±1.36 mM, 15.31±1.57* mM and 7.58±0.69^#^ mM (*, P<0.05 vs. wistar rats and ^#^, P<0.05 vs. GK-Saline) respectively. At the end of treatment period, no significant difference was also observed between the body weights of GK-Saline vs. GK-Gel.

### IL-1Ra Loaded in PF127 Gel Improves Glucose Tolerance in GK Rats

**Figure 2:**
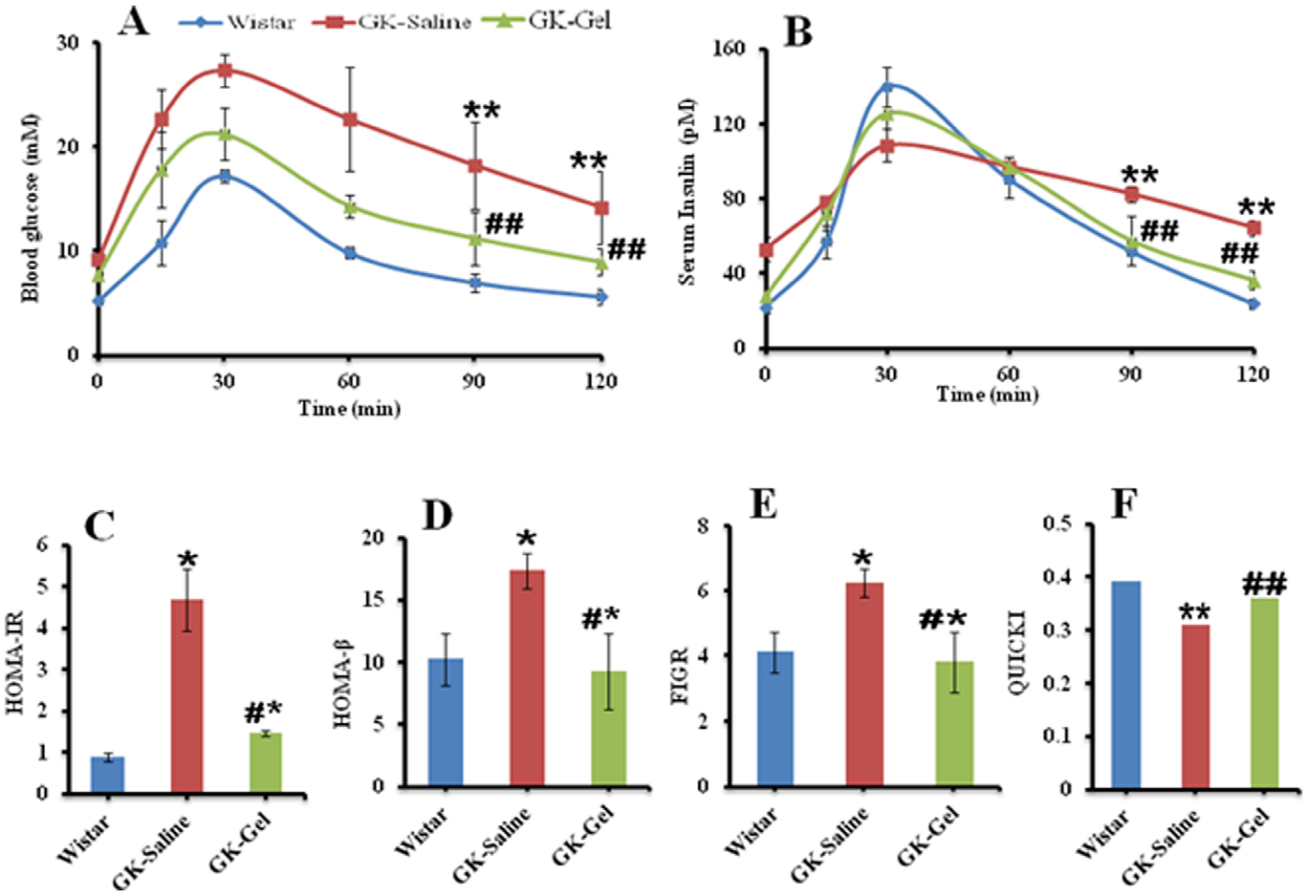
Intraperitoneal glucose tolerance test. The time courses of serum levels of (A) glucose and (B) insulin are expressed as mean ± SD. (C) HOMA-IR, (D) HOMA-β, (E) FIGR and (F) QUICKI were also calculated by using the values of serum levels of glucose and insulin. n = 4 for wistar rats, n = 5 for both groups of GK rats. ^*^, P<0.05; ^**^, P<0.01; compared to wistar rats groups. ^#^, P<0.05; ^##^, P<0.01 compared to GK-Saline group.

IPGTT was performed to verify the ability of IL-1Ra loaded in PF127 gel to induce insulin secretion in response to glucose administration. During IPGTT, GK-Saline exhibited significantly (P<0.05) high glycemic values before (0 min) and after the glucose administration as compared to the values of wistar and GK-Gel animals evaluated at all time points. In case of GK-Gel, although, the values of glucose before (0 min) and after glucose administration were high as compared to wistar rats at all time points but significantly (P<0.05) decreased peak glucose levels were observed at each time points when compared to GK-Saline (Fig. 2A). After 120 minutes, the plasma glucose levels in GK-Saline were found to be significantly high as compared to GK-Gel and wistar groups. In IPGTT, no significant difference (P<0.01) was found between the basal plasma glucose levels of wistar and GK-Gel from the plasma glucose levels observed after 120 minutes of glucose administration whereas, a highly significant difference (P<0.001) was observed between the basal plasma glucose levels and plasma glucose levels after 120 minutes of glucose administration (Fig. 2A) in GK-Saline.

During IPGTT, the serum levels of insulin for these groups were also measured at all time points (Fig. 2B). In GK-Saline, the serum level of insulin was 1.5-folds high as compared to wistar rats and 0.9-folds from GK-Gel before (0 min) the administration of glucose. After glucose administration, wistar rats underwent high serum insulin levels (6-folds from their baseline fasted serum insulin levels) in response to glucose after 30 minutes whereas, serum levels of insulin in GK-Gel were increased 3.7-folds as compared to their baseline fasted serum insulin levels. The serum level of insulin did not significantly increase (just 1-fold increased as compared to their base line fasted insulin levels) in GK-Saline. Wistar rats exhibited 2.1-fold lower fasted serum insulin levels (P<0.05) before (0 min) and 1.24-fold increased serum insulin levels as compared to GK-Saline after 30 minutes of glucose administration. GK-Gel group exhibited significantly lower fasting serum insulin levels (0.9-fold; P<0.05) before (0 min) and increased serum insulin levels (1.21-fold) as compared to GK-Saline after 30 minutes of glucose administration. Compared with wistar and GK-Gel group, serum levels of insulin in GK-Saline before (0 min) and at 120 minutes of glucose administration were significantly (P<0.05) high (Fig. 2B).

### Insulin Sensitivity Determinations

As we observed the protective therapeutic effects of IL-1Ra loaded in PF127 gel on blood glucose levels, glucose tolerance and insulin secretion during the IPGTT, we further elucidated these effects by analyzing the insulin resistance, β-cell function to secrete insulin and sensitivity with calculating HOMA-IR, HOMA-β, FIGR and QUICKI. These parameters were based on the fasting levels of glucose and insulin (IPGTT). The HOMA-IR value (Fig. 2C) for GK-Saline was 4.28-fold (P<0.05) high as compared wistar rats and 2.3-fold (P<0.05) high when compared with GK-Gel group. HOMA-IR value for GK-Gel showed that IL-1Ra loaded in PF127 may increase the insulin sensitivity in GK-Gel group. The β-cell function to secrete insulin was assessed by HOMA-β and the HOMA-β value for GK-Saline was 0.7-fold (P<0.01) and 0.8-fold (P<0.05) high when compared with wistar and GK-Gel group respectively (Fig. 2D). Surprisingly, there was no significant difference in HOMA-β between wistar and GK-Gel group. Similar results were also observed when we calculated FIGR (Fig. 2E). Subsequently, we calculated QUICKI for these groups and a highly significant difference for GK-Saline was observed when compared with wistar (P<0.01) and GK-Gel (P<0.001) whereas, there was not a significant difference observed between the QUICKI values of wistar and GK-Gel group animals (Fig. 2F).

### Effect of IL-1Ra Loaded in PF127 Gel on Metabolic Parameters

**Figure 3:**
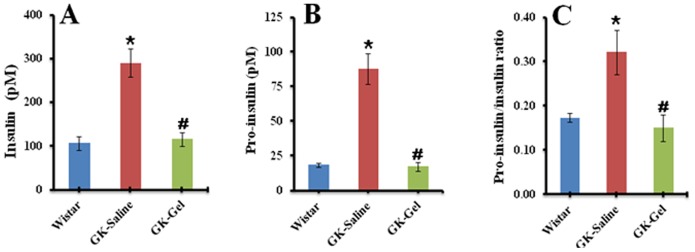
At the end of treatment, serum levels of (A) fed insulin, (B) pro-insulin and (C) pro-insulin/insulin ratio. ^*^, P<0.05; compared to wistar rats groups. ^#^, P<0.05; compared to GK-Saline group. n = 4 for wistar rats, n = 5 for both groups of GK rats.

At the end of one month treatment, rats were killed mercifully and their serum was separated from collected blood samples by centrifugation. The metabolic parameters analyzed are summarized in Table 1. The body weights of GK-Saline and GK-Gel were found to be significantly lower (P<0.05) than that of wistar group. GK-Saline exhibited significant hyperglycemia [36] as compared to wistar and GK-Gel (P<0.05) whereas, due to continuous sustained delivery of IL-1Ra from PF127 gel, we did not find any significant difference between the fed blood glucose levels of wistar and GK-Gel group animals (Table 1). Sustained delivery of IL-1Ra also revealed its great therapeutic potentials to decrease circulating levels of insulin and pro-insulin on GK rats. At the end of treatment, a significant difference (P<0.05) was observed between circulating levels of insulin, pro-insulin and pro-insulin/insulin ratio for GK-saline and GK-Gel group animals (Fig. 3A, B and C respectively) whereas, we did not observe any significant difference between these parameter in GK-Gel vs. wistar group animals. We also measured various lipid profile biomarkers (FFAs, TGs, TC, HDLs, LDLs and VLDLs) to predict the effect of IL-1Ra through sustained delivery from PF127 gel. The circulating levels of these parameters were significantly reduced in GK-Gel group when compared with GK-Saline (P<0.05) whereas, a highly significant difference was also observed in Gk-Saline group when compared with wistar group animals (Table 1). We also calculated TC/HDLs, TGs/HDLs and LDLs/HDLs ratios among the same group animals. A highly significant difference (P<0.01) was observed when TC/HDLs, TGs/HDLs and LDLs/HDLs ratios of GK-Saline were compared with that of wistar and GK-Gel separately (Table 1). We also calculated a systemic inflammatory marker (IL-6) in all groups. The values of IL-6 in GK-Saline group were significantly (P<0.05) high as compared to wistar and GK-Gel group animals whereas, a non significant difference was observed between the serum levels of IL-6 in GK-Gel vs. wistar group animals (Table 1). Conclusively, sustained delivery of IL-1Ra loaded in PF127 gel showed the ability to prevent hyperinsulinaemia, hyperlipidemia and elevated levels of IL-6 in GK rats.

**Table 1:** Metabolic parameters for age-matched male GK and non-diabetic wistar rats measured at the end of one month treatment.

Parameters	Animal groups		
	Wistar	GK-Saline	GK-Gel
Body weight (gm)	264.6±21.67	223±24.20*	228±23.11*
**Glycemic control biomarkers**			
Glucose (mM)	7.08±1.36	15.31±1.57*	7.58±0.69^#^
Insulin (pM)	105.56±15.29	289.52±31.91*	114.77±15.37^#^
Pro-insulin (pM)	18.19±1.64	87.76±11.18*	17.15±3.07^#^
**Lipid profile biomarkers**			
FFAs (mM))	0.48±0.08	0.85±0.17*	0.52±0.05^#^
TGs (mM))	1.24±0.12	3.85±1.16*	1.44±0.32^#^
TC (mM))	1.81±0.06	4.79±1.22*	2.13±0.12^#^
HDLs (mM))	0.92±0.11	2.48±0.86*	1.16±0.09^#^
TC/HDLs	1.97±0.00	1.93±0.00**	1.84±0.00^##^
TGs/HDLs	1.35±0.00	1.55±0.00**	1.24±0.00^##^
LDLs (mM))	0.47±0.02	2.23±0.53*	0.77±0.11^#^
LDLs/HDLs	0.51±0.00	0.90±0.00**	0.66±0.00^##^
VLDLs (mM))	0.42±0.02	1.44±0.46*	0.56±0.04^#^
**Inflammatory Biomarker**			
IL-6 (pg)	321.43±18.63	850±55.69*	340±49.07^#^

All parameters were measured in serum under fed condition. FFAs: free fatty acids, TGs: triglycerides, TC: total cholesterol, HDLs: high density lipoproteins, LDLs: low density lipoproteins, VLDLs: very low density lipoprotein, IL-6: interleukin-6. IL-1Ra: interleukin-1 receptor antagonist.

* , *p*<0.05; **, *p*<0.01 versus age-matched wistar group.

#, *p*<0.05; ##, *p*<0.01 versus GK-saline group as determined by student *t*-test. values are given as mean ± SD for the group of 5 animals each.

### Effect of IL-1Ra and PF127 on Kidney Function of GK Rats

At the end of treatment period, we observed the kidney functions for all groups. We measured urea nitrogen, creatinine and uric acid in serum. The serum levels of urea nitrogen, creatinine and uric acid in GK-Saline groups were significantly (P<0.05) higher as compared to that of wistar group (Table 2) whereas, sustained delivery of IL-1Ra loaded in PF127 gel significantly suppressed the levels of urea, creatinine and uric acid in GK-Gel group near to the serum levels of observed in wistar group (Table 2). We collected 24 hr urine from all rats in metal-free propylene tubes to measure the pH value, urinary albumin and creatinine. We also calculated the ACR from these measured values. GK-Saline groups excreted a large volume of urine with dark brown color in 24 hrs as compared to wistar and GK-Gel group (P<0.001). The pH value of GK-saline was also high when compared with wistar group (Table 2). GK-Saline group exhibited a significant increase in urinary albumin, creatinine, and ACR when compared with wistar group animals (P<0.01). In case of GK-Gel group animals, IL-1Ra loaded in PF127 gel exhibited dramatic effects on urinary markers. A highly significant reduction (P<0.01) of urinary albumin, creatinine, and ACR was observed in GK-Gel group animals representing the non toxic effects of PF127 on kidneys (Table 2). Therefore, sustained delivery of IL-1Ra significantly suppressed the development of albuminuria in GK rats in accordance with reflecting the safe use of PF127.

**Table 2:** Estimation of kidney function for age-matched male GK and non-diabetic wistar rats measured at the end of one month treatment.

Parameters	Animal groups		
	Wistar	GK-Saline	GK-Gel
**Serum Markers**			
BUN (mM))	4.6±1.30	7.16±1.37*	4.86±0.58^#^
Creatinine (µM))	33.8±2.77	43.68±5.22*	32.2±2.39^#^
UA (µM))	129.8±16.56	181.75±26.33*	122.56±25.16^#^
**Urinary Markers**			
Volume of urine (ml/day)	15.50	30.00*	18.00^#^
Color	Light yellow	Dark yellow	Light yellow
pH	6.5	8.5**	6.8^##^
Microalbumin (mg/L)	8.12	28.24**	9.49^##^
Creatinine (g/L)	0.92	2.69	1.07
ACR	7.96	10.50**	8.11^##^

Serum parameters were measured under fed condition. Urinary parameters were measured after the collection of urine for 24 hrs. BUN: blood urea nitrogen, UA: uric acid, ACR: albumin to creatinine ratio.

* , *p*<0.05; **, *p*<0.01 versus age-matched wistar group.

#, *p*<0.05; ##, *p*<0.01 versus GK-saline group as determined by student *t*-test. values are given as mean ± SD for the group of 5 animals each.

### Immunohistochemical Analysis of CD68 in Pancreatic Islets

**Figure 4:**
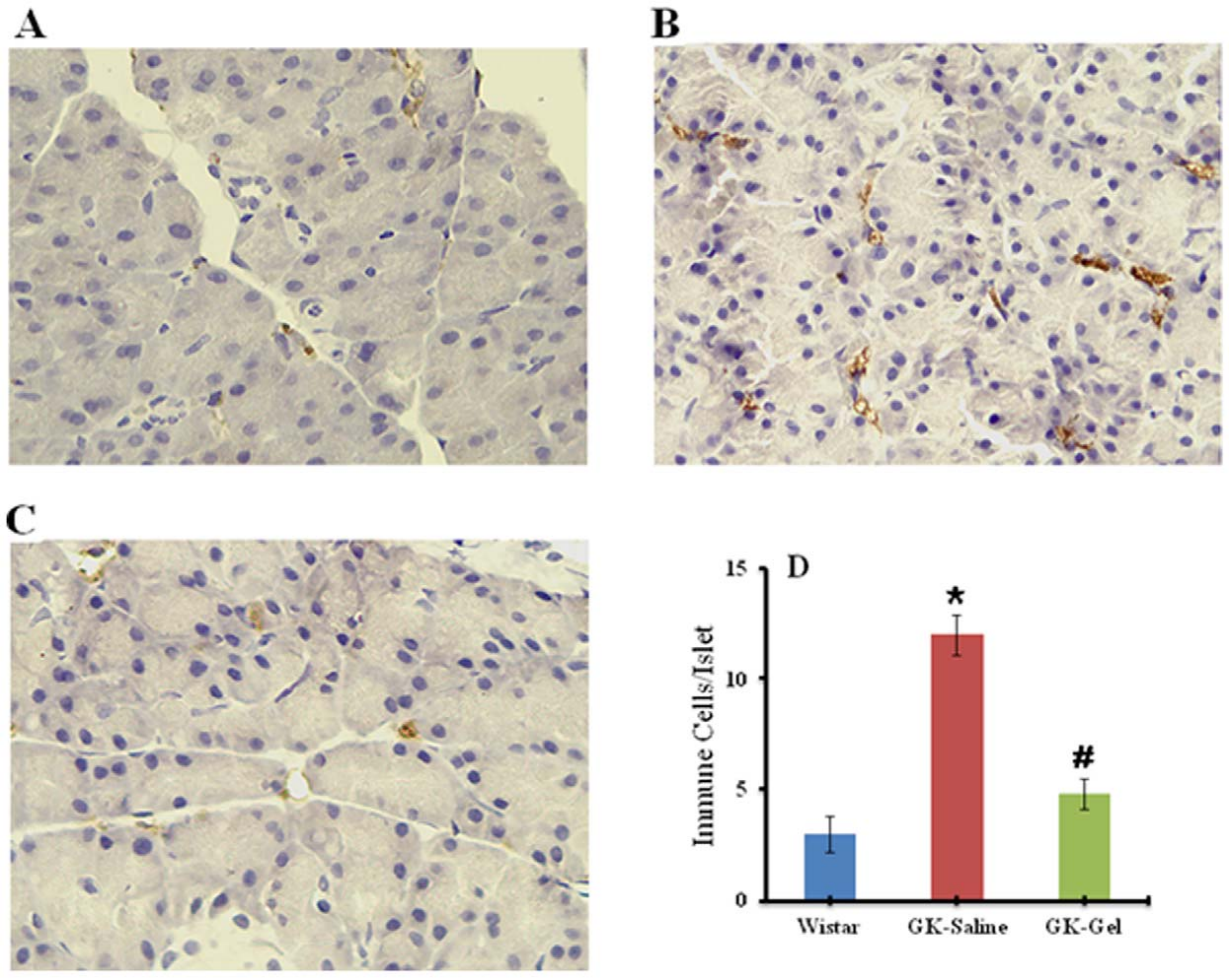
Immunohistochemical staining of macrophage infiltration marker (CD68) in pancreatic islets (IHC ×400) of: (A) Wister rat (B) GK-Saline group showing abundant CD68 labeling representing rich macrophage infiltration (C) GK-Gel group showing normal organization of islets with minimal macrophage infiltration. (D) Quantification of immune cells/pancreatic islets by immunohistochemistry. ^*^, P<0.05; compared to wistar rats groups. ^#^, P<0.05; compared to GK-Saline group. n = 3 for wistar rats, n = 4 for both groups of GK rats.

To investigate the expression of inflammation in pancreatic islets, immunohistochemical analysis was performed for CD68, taking macrophage infiltration as marker (Fig. 4). The islets of GK rats treated with IL-1Ra loaded in Gel represented significantly reduced CD68 labeling indicating minimal macrophage infiltration in islet (Fig. 4C). In addition, an overexpression of the CD68 was observed in GK-Saline group (Fig. 4B) islets advocating that IL-1Ra treatment might have reduce the inflammatory responses in IL-1Ra-treated GK islets most probably by decreasing the immune cell infiltration in the GK rats treated with IL-1Ra loaded in PF127 gel (Fig. 4D).

### Histopathological Inspection of Skin Sections

**Figure 5:**
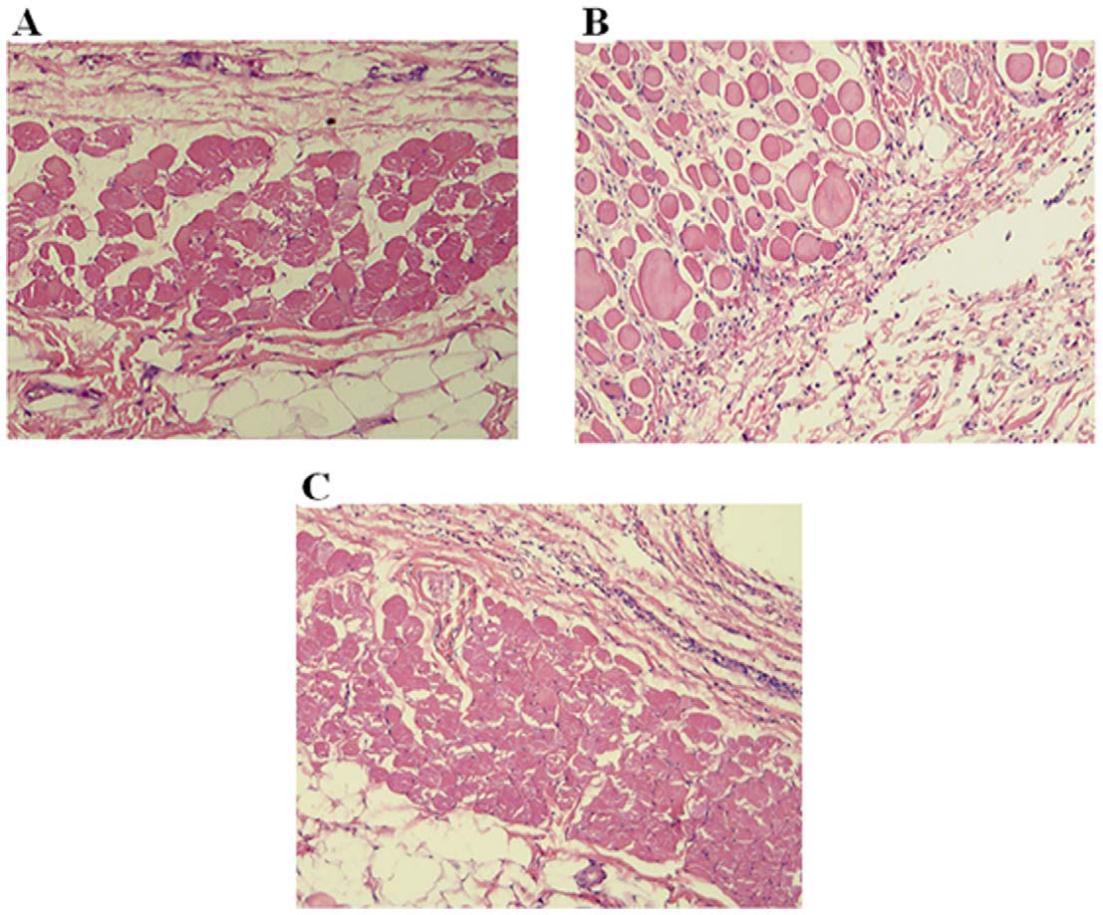
Histological micrographs of hematoxylin-eosin stained skin section (H&E×100) showing normal subcutaneous tissue of: (A) Wister rat group (B) GK-Saline group (C) GK-Gel group. n = 3 for wistar rats, n = 4 for both groups of GK rats.

The pathological examination of histological sections (H&E×100) of skin from the site of injection was carried out at the end of treatment to investigate any sign of inflammatory reactions. As shown in Fig. 5, no appearance of macrophages or lymphocytes were observed at the site of injection depicting no significant inflammation in the surrounding tissues at the site of injection suggesting a safe administration of PF127 via subcutaneous injections.

### Histopathological Inspection of Kidney Sections

**Figure 6:**
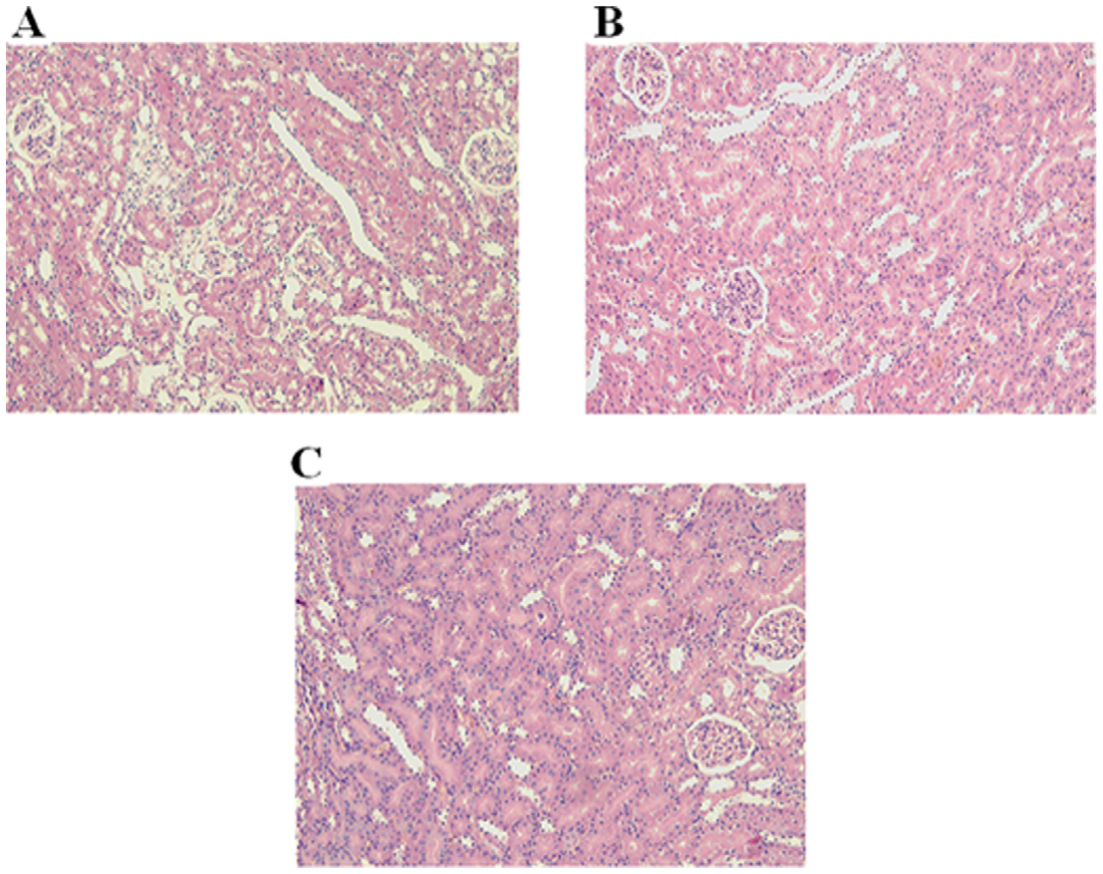
Histological micrographs of hematoxylin-eosin stained kidney section (H&E×100) of: (A) Wister rat showing normal glomeruli and tubules, (B) GK-Saline group showing normal appearance of tubules, lumen and glomeruli (C) GK-Gel group showing normal renal tissue, tubules, lumen and glomeruli. n = 3 for wistar rats, n = 4 for both groups of GK rats.

The histopathological inspection of kidney section (H&E×100) revealed normal glomeruli and tubules of Wister rat (Fig. 6A). The appearance of GK-Saline rat’s kidney also showed normal tubules, lumen and glomeruli (Fig. 6B). Interestingly, the kidney of GK-Gel rats depicted no pathological changes in the renal tissue, tubules, lumen and glomeruli (Fig. 6C) suggesting that PF127 had no significant damaging effects on the excretory organ.

### Adverse Events

We did not observe any significant changes in body weights of GK rats treated with Gel versus GK rats treated with saline. No rat died due to IL-1Ra and/or PF127 related adverse events. In particular, we also did not observe the acute hypoglycemia in treated group of GK rats. No significant tissue necrosis, hemorrhaging, hyperemia, edema, or muscle damage was observed throughout our examinations.

## Discussion

In recent years, research has proved that T2DM is a chronic auto-inflammatory syndrome [3], [4] in which IL-1β pays its pivotal role for initiation of various inflammatory mechanisms for dissemination of T2DM [9], [43]. IL-1Ra is naturally occurring anti-inflammatory antagonist of IL-1β that has recently been approved from FDA for the treatment of T2DM but due to its short half-life, higher dose with frequent dosing intervals are required. In our previous work, we developed a new dosage form for IL-1Ra using three different concentrations of PF127 (20, 25 and 30%) and investigated its in-vitro and in-vivo effects [26]. Among these three concentrations, 25% PF127 gel formulation was found to be the best one that prolonged the *in vitro* and *in vivo* release, showed greater efficacy to induce hypoglycemia and inhibited IL-1β-stimulated production of IL-6 in wistar rats when compared with that of IL-1Ra solution. 25% PF127 gel significantly sustained the plasma concentration of IL-1Ra for longer period of time as compared to 20% PF127 gel and/or pure IL-1Ra solution in wistar rats. The maximum plasma concentration (C_max_) of IL-1Ra loaded in 25% PF127 gel was achieved at 4 hr whereas the C_max_ for pure IL-1Ra solution was achieved within 15 minutes. Due to sustained release of IL-1Ra from 25% PF127 gel, the plasma half-life of IL-1Ra was significantly high (12.53 hr) when compared directly with that of pure IL-1Ra solution. Based on the sustained release effects of IL-1Ra loaded in 25% PF127 gel, here in our present work, we extended this approach using diabetic GK rats that genetically inbreeded from wistar rats. We administered IL-1Ra loaded in PF127 gel for one month into GK rats. The optimum dose (10 mg/kg) was standardized and confirmed by previous study [19]. Our present investigations indicate that IL-1Ra loaded in PF127 gel exhibited its sustained hypoglycemic and protective effects on treated animals. During the treatment period, we observed a significant reduction of plasma glucose in treated animals when compared (P<0.05) with GK-Saline (Fig. 1A). Our results are highly supported by already published reports [19], [22], [44]. The continuous sustained delivery of IL-1Ra released from PF127 gel demolished the deleterious effects of glucose by blocking pro-inflammatory signaling of IL-1β [16], [23] resulted in increased β-cell proliferation. Previous studies show that IL-1Ra has the ability to recover β-cell survival and augment glucose-stimulated insulin secretion in diabetic GK rats [19] rats fed with high fat diet [16] and human pancreatic islets [22], and improves insulin sensitivity in peripheral tissues [19].

Augmented plasma levels of glucose destroy the secretory functions of β-cells by inducing secretion of IL-1β in pancreatic islets [4] that not only impairs the β-cell function and induce apoptosis but also cause the insulin resistance in peripheral tissues. In diabetic GK rats, glucose intolerance is increased after post weaning [37]. Our IPGTT results show that sustained delivery of IL-1Ra increased the glucose tolerance (Fig. 2A), insulin secretion (Fig. 2B) in GK-Gel group when compared with GK-saline group. Interestingly, improved glucose tolerance in GK-Gel group is strongly correlated with increase insulin secretion in response to administered glucose suggesting the role of pro-inflammatory mechanisms in glucose dyshomeostasis and our present results are in accordance with already published reports. [22], [38], [44].

Insulin resistance is a hallmark for the pathogenesis of T2DM and homeostasis model assessment (HOMA) and QUICKI are widely used to quantify the insulin resistance and β-cell secretory function [42], [45]–[47]. These two mathematical formulas are differed only in their normalization factor i.e. constant denominator in HOMA and log-transformation in QUICKI. These formulas are most similar to the biological phenomenon for insulin resistance and their reproducibility solely depends on the fasting levels of glucose and insulin used to calculate their values [48]. By using HOMA, we assess that decrease in β-cell function to secrete insulin is represented by failure of β-cell response to plasma glucose concentrations. In our present study, IL-1Ra loaded in PF127 gel sufficiently improved β-cell secretory function and decreased insulin resistance in response to exogenously administered glucose into GK-Gel group (Fig. 2C and D). Our results are in accordance with already published reports [19], [45]. The HOMA-IR, HOMA-β, FIGR and QUICKI values (Fig. 2C,D,E and F respectively) from these three groups strongly correlate the results of IPGTT independently and are in accordance with our previous results [44].

β-cell’s dysfunction directly correlates with islet’s inflammation [13], [15] that may cause the impaired insulin secretion from Β-cells and increased insulin resistance in peripheral tissues. Due to broad spectrum anti-inflammatory effects, IL-1Ra exhibits its dual therapeutic potentials. First, it protects β-cells of pancreatic islets in insulin processing and/or signaling from direct cytotoxic effects of IL-1β and secondly, it also blocks IL-1β-induced other cytokines and chemokines in pancreatic islets [19]. Our present study shows that sustained delivery of IL-1Ra from PF127 gel not only reduced the extent of hyperglycemia in GK-Gel group (Fig. 1A) but also improved the secretory function of β-cells for insulin processing (as evidenced from the decreased values of pro-insulin/insulin ratio (Fig. 3C) and HOMA-β (Fig. 2D) in GK-Gel group when compared with GK-saline) and insulin sensitivity (as evidenced from decreased HOMA-IR and increased QUICKI values for GK-Gel group (Fig. 2C and F) when compared with GK-saline group).

Hyperglycemia and hyperlipidemia play their permissive role to trigger the secretion of IL-1β from pancreatic islets and cause dysfunction of β-cells in diabetic GK rats [23], [49], [50]. Due to decreased β-cell dysfunction, GK rats further develop increased levels of TGs, FFAs, TC, HDL and/or TC/HDL ratio [19], [44], [47], [51] and impairs glucose induced insulin secretion from pancreatic islets [19], [44], [50]. Increased circulating levels of lipid profiles may induce the stimulation of pro-inflammatory cytokines and/or chemokines from endothelial cells and vascular smooth muscles resulting in an increased oxidative stress [44]. These inflammatory responses move around β-cells of pancreatic islets and produce further pro-inflammatory mediators that might deteriorate β-cells [4], [9], [43]. IL-1Ra binds to IL-1RI β-cells with high affinity on without triggering any response and prevents the binding of IL-1β. High levels of lipid profiles (Table 1) are suppressed by the continuous delivery of IL-1Ra from PF127 gel after one month treatment in which insulin secretion was increased as verified by serum levels of insulin measured during IPGTT (Fig. 2B), HOMA-β (Fig. 2D). Similar types of findings in which IL-1Ra decreased lipid profiles have also been reported elsewhere [19], [44].

Diabetic nephropathy is one of the major morbidity and mortality factors that are most commonly confronted by diabetic patients that usually lead to end-stage renal failure. In clinical practice, one of the major adverse effects of anti-diabetic agent is nephrotoxicity that frequently leads to acute renal failure. Animal models are used to investigate the desired therapeutic and undesired cytotoxic effects as it is quite essential because renal damage would alter the structure and function of kidneys that might have serious effects on overall metabolism of the body. BUN a metabolic product of protein metabolism, UA a product of purine nucleotides and creatinine are considered as biomarkers for renal function in diabetic nephropathy [52]. The increased concentrations of BUN, UA and creatinine with increased urine output might represent the renal failure in diabetes mellitus [53], [54]. Treatment with IL-1Ra loaded in PF127 gel reversed these parameters near to the normal values of wistar rats (Table 2) that could be due decreased metabolic disturbances of proteins as evidenced by improved glycaemic control during IPGTT (Fig. 2). Our results are highly supported by already published reports [55], [56]. Diabetic nephropathy is a clinical manifestation of developed microalbuminuria that may be due to impaired tubular reabsorption and/or leakage of albumin due to injured glomeruli [57] that may leads to alteration of selective barriers of glomeruli. Urinary albumin and creatinine have been considered as defensive biomarkers for diagnosis of diabetic nephropathy [58]. Abnormally, elevated levels of these may cause the renal failure [59]. Here in our study, we report that in GK-Saline group, the values of ACR were significantly (p<0.01) high as compared to wistar rats and/or GK-Gel group whereas, IL-1Ra loaded in PF127 gel suppressed this effect that were consistent with already published reports elsewhere [60]–[63].

Recently, inflammation has attained considerable attention for pathogenesis of T2DM [3], [4], [64] in which many inflammatory mechanisms and responses are involved [5]. Inflammatory responses may characterize the presence of various pro-inflammatory mediators (cytokines and chemokines), immune cells and macrophage infiltrations, amyloid deposits, apoptotic cells and fibrosis. Therefore, it is essential to modulate the intra-islet inflammatory responses. IL-1Ra may have the potential to suppress these inflammatory mechanisms and responses in pancreatic islets [3], [13], [19], [65]. In our data, IL-1Ra released from PF127 gel could sufficiently reduce islet inflammation in GK-Gel group by reducing the expression of macrophage infiltration marker CD68 (Fig. 4) and our results are in agreements with already published reports elsewhere [13], [19], [65].

Interesting and important finding of our present research is that we did not find any alterations in the normal histology of skin and kidneys due to the continuous administration of PF127 for one month in GK-Gel group. We also estimated the normal functions of kidneys by measuring various kidney function markers and surprisingly we did not noticed any significant changes of kidney function markers when compared with that of wistar rats. To the best of our knowledge, it is noteworthy that no prior studies have been ever been conducted in which such type of findings have observed showing the safe use of PF127 for sustained delivery of any proteinous drug such as IL-1Ra continuously for one month. Here for the first time, we report that PF127 is safe and may not alter the normal physiological functions of the body and prolong therapeutic potentials of IL-1Ra through its sustained delivery.

Here in our present study, we have also compared the therapeutic efficacy of IL-1Ra loaded in PF127 gel with our already published results of IL-1Ra solution on GK rats [44]. The comparison of therapeutic efficacy of IL-1Ra in different dosage forms is elaborated in Table 3. In our present work, the dose of IL-1Ra loaded in PF127 gel was 10 mg/kg/day whereas for IL-1Ra solution it was 20 mg/kg/day. From the results of table 3, it has been clearly found that although, the dose of IL-1Ra loaded in PF127 gel was 50% less than that of IL-1Ra solution but therapeutic efficacy of IL-1Ra loaded in PF127 gel was significantly high to revert the symptoms of hyperglycemia and hyperlipidemia in diabetic GK rats as compared to IL-1Ra solution.

**Table 3:** Comparison of therapeutic effects between IL-1Ra loaded in PF127 gel and IL-1Ra solution in GK rats.

Parameters	Therapeutic effects of IL-1Ra on metabolic parameters in GK rats	
	IL-1Ra loaded in PF127 gel^a^	IL-1Ra solution^b,^ *
Glucose (mM)	7.58±0.69	8.61±0.69
Insulin (pM)	114.77±15.37	230±15.37
Pro-insulin (pM)	17.15±3.07	18.90±3.07
TGs (mM)	1.44±0.32	2.05±0.32
TC (mM)	2.13±0.12	2.85±0.12
HDLs (mM)	1.16±0.09	1.85±0.09
BUN (mM)	4.86±0.58	6.45±0.58
Creatinine (µM)	32.2±2.39	47.18±2.39
UA (µM)	122.56±25.16	174.75±25.16

Therapeutic effects of IL-1Ra (either loaded in PF127 gel or in solution form) in GK rats were compared. TGs: triglycerides, TC: total cholesterol, HDLs: high density lipoproteins, BUN: blood urea nitrogen, UA: uric acid.

a, 10 mg/kg/day.

b, 20 mg/kg/day.

* , For detailed description, see Akash et al [44].

From various previous studies, it has been proved that PF127 is safe for the sustained delivery of various proteins such as IL-2 [31], insulin [29], [32], recombinant hirudin [33], oxytocin [34] and lysozyme [35], and prolongs the stability of proteins [26]–[29]. From clinical perspective, our investigations that are derived from an experimental protocol could stimulate further clinical trials on type 2 diabetic patients to expedite therapeutic uses of PF127 for safe and effective delivery of IL-1Ra that exhibit its sustained release effects devoid of altering normal physiological functions of body and protecting the efficacy of encapsulated IL-1Ra till its delivery to the target site.

### Conclusion

In conclusion, our findings offer better understandings of safe and effective use of biodegradable polymer PF127 for continuous sustained delivery of IL-1Ra up to one month through subcutaneous route. Our findings also exhibit that PF127 prolonged therapeutic potentials of IL-1Ra without altering normal physical functions of the body. The protective use of PF127 for sustained delivery of IL-1Ra may explain long-lasting effects of IL-1Ra using single dose of IL-1Ra in GK rats despite any kind of adverse events to the body without altering the normal functions of kidneys. Our data also highlight the potential of role of biodegradable polymer PF127 for proficient delivery of IL-1Ra. Therefore, counteracting the short biological half-life of IL-1Ra using PF127 for its sustained delivery is one way to treat T2DM.
